# Antifungal and genotoxic effects of *Thymus serpyllum* as a root canal irrigant

**DOI:** 10.1002/cre2.837

**Published:** 2024-02-06

**Authors:** Ariana Kameri, Agime Dragidella, Arben Haziri, Zeqir Hashani, Kemajl Kurteshi, Arsim Kurti

**Affiliations:** ^1^ Department of Dental Pathology and Endodontics, Faculty of Medicine University of Prishtina “Hasan Prishtina” Prishtina Kosova; ^2^ Department of Chemistry, Natural Science Faculty University of Prishtina “Hasan Prishtina” Prishtina Kosova; ^3^ Faculty of Education University “Fehmi Agani” Gjakove Kosova; ^4^ Department of Biology, Natural Science Faculty University of Prishtina “Hasan Prishtina” Prishtina Kosova; ^5^ Department of Microbiology, Medical Faculty University of Prishtina “Hasan Prishtina” Prishtina Kosova

**Keywords:** *Candida Albicans*, herbal extract, root canal

## Abstract

**Objectives:**

The aim of this study was the assessment of the efficiency of the ethyl acetate (EthOAc) extract of *Thymus serpyllum* against *Candida albicans* and to compare it with sodium hypochlorite (NaOCl) and chlorhexidine (CHX), as well as their genotoxic effect.

**Material and Methods:**

The antifungal effectiveness of the EthOAc extract of *Thymus serpyllum* was determined using the agar disk diffusion method. The inhibition zones induced by the EthOAc extract were compared after 5 min, 60 min, and 24 h to those induced by standard solutions (2% CHX and 2% NaOCl). An in vitro genotoxicity assay was performed in cultured lymphocytes from the blood of human volunteers to observe micronuclei formation. Statistical analysis of the results was performed using the Kruskal–Wallis test and one‐way analysis of variance.

**Results:**

The inhibition zone of combination of CHX with EthOAc extract of *Thymus serpyllum* against *C. albicans* was 29.7 mm after 5 min, 28.3 mm after 60 min, and 29 mm after 24 h. The inhibition zone of NaOCl in combination with EthOAc extract of *Thymus serpyllum* against *C. albicans* was 0 mm. The EthOAc extract of *Thymus serpyllum* did not show a genotoxic effect on lymphocyte cells.

**Conclusions:**

The EthOAc extract of *Thymus serpyllum* in combination with CHX may be a useful root canal disinfection in endodontic therapy.

## 1 INTRODUCTION

Adequate mechanical preparation and chemical disinfection of a root canal is the primary way to eradicate microorganisms and their by‐products, which helps to prevent periapical pathologies (Nair, 2004). Several solutions are used to irrigate the root canal. These solutions should have low toxicity, break down tissue and debris, have antibacterial properties, inactivate endotoxins, lubricate, remove the smear layer, not be caustic to periodontal tissues, and should not cause anaphylactic reactions (Rahimi et al., 2014).

Sodium hypochlorite (NaOCl, used at a concentration of 0.50%–5.25%) is the most common irrigant used in endodontics. In addition to its antibacterial action, this irrigant also enables the mechanical removal of debris and breaks down pulp and collagen residues. NaOCl is the only irrigant that dissolves vital and necrotic organic tissue (Sousa et al., 2005; Xu et al., 2022). Combining NaOCl with ethylenediaminetetraacetic acid or citric acid completely removes the smear layer (Sahebi et al., 2016). Although NaOCl has many benefits, there are some undesirable properties as well, such as its allergic and/or toxic potential and bad taste (Spanberg et al., 1973; Zehnder, 2006).

Chlorhexidine (CHX, used at a concentration of 0.2%–2.0%) is an irrigant that has been used in endodontics since 1940 thanks to its broad antimicrobial activity (Mohammadi et al., 2014; Tomás et al., 2011; Vianna et al., 2004). CHX penetrates the cell wall and the plasma membrane of bacteria and fungi, respectively, to enter the cytoplasm. In high concentrations, CHX coagulates intracellular components (Cieplik et al., 2019; Gao et al., 2018).

Unlike NaOCl, CHX does not have a bad taste and has been shown to be less toxic and does not irritate periapical or periradicular tissues (Gomes et al., 2013). However, unlike NaOCl, it is unable to break down tissues (Okino et al., 2004) or dissolve necrotic tissue remnants (Surender et al., 2017) and the smear layer (Dewi, 2020). CHX is more effective for gram‐positive bacteria than for gram‐negative bacteria (Rusu et al., 2017). It can be used in combination with Ca(OH)_2_ as an intracanal medicament (Jacques Rezende Delgado et al., 2013) in necrotic tissue, retreatment cases, and in vital pulp (Turk et al., 2009). However, the antimicrobial substantivity of CHX preparations delay microleakage into the root canal (Athanassiadis et al., 2007).

Because of the complexity of the root canal system, endodontic therapy can fail, regardless of which irrigation techniques and instruments are used (Alfirdous et al., 2021; Fezai & Al‐Salehi, 2019). Because biofilms prefer moist environments, secondary intraradicular infections occur as a result of microorganism proliferation during endodontic therapy, especially in cases where the endodontic space is left open, when displacement of the temporary filling between sessions leads to coronal penetration, or when there are defects in the crown fillings (Siqueira et al., 1999). For example, it has been shown that *Candida albicans* can be isolated in 1%–17% of cases of infected canals (Waltimo et al., 1999; Waltimo et al., 2004). *Candida albicans* is commonly studied by researchers, because it is a prevalent yeast that can cause periradicular infections.

*Candida albicans* is known as intratubular microorganisms that may be the primary cause of microbial resistance to root canal treatment and post‐treatment recurrence of periradicular lesions (Yoo et al., 2020).

Current endodontic drugs are limited in their ability to eliminate microorganisms from the root canal because of microbial resistance (Wong et al., 2021). Furthermore, these drugs also have possible cytotoxic and genotoxic reactions. To address these concerns, application of plant extracts with medicinal properties has gained recent attention (Amirifar et al., 2022; Bolouri et al., 2022; Jain et al., 2022). Additional benefits of these natural extracts include their availability, reasonable cost, and low toxicity, as well as a lack of microbial resistance and increased rate of action compared with those of currently used drugs (Khodaei et al., 2021). Together, this improves the cost effectiveness of treating various dental diseases (Moghadam et al., 2020).

One extract that has been shown to be effective in treating *C. albicans* is *Thymus vulgaris* oil that is derived from *Thymus serpyllum* from the Lamiceae family (Fani & Kohanteb, 2017), which grows in the mountains of Sharr in Kosovo. These species are also usually found in soils with siliceous and limestone substrates in meadows and hills. To use plant extracts as an alternative therapy in dentistry, and especially in endodontics, the extract should have antimicrobial effects but also have minimal genotoxic and cytotoxic effects. The latter is important because the extract will be in contact with the periapical tissue, which is often irritated after endodontic treatment (Jain & Ranjan, 2014). Therefore, to find an ideal endodontic medication, antimicrobial, genotoxic, and cytotoxic effects must be evaluated to investigate their impact on vital tissues (Karkehabadi et al., 2018).

The aim of this study was to compare the fungicidal properties of *T. serpyllum* ethyl acetate extracts against *C. albicans* with those of NaOCl and CHX, as well as to evaluate its genotoxic effects.

## 2 MATERIAL AND METHODS

This study was approved by the Ethical Committee of the Faculty of Medicine‐Dentistry branch in University of Prishtina “Hasan Prishtina” (protocol number 2417), and patients signed a written informed consent form before the treatment.

The materials used in this study included 2% NaOCl (ChloraXD, Cerkamed) titrated with sodium thiosulfate solutions, 2% CHX (Gluco‐Chex, Cerkamed), ethyl acetate (EthOAc; Sigma Aldrich), and dimethylformamide (DMF; Sigma Aldrich).

**Figure 1:**
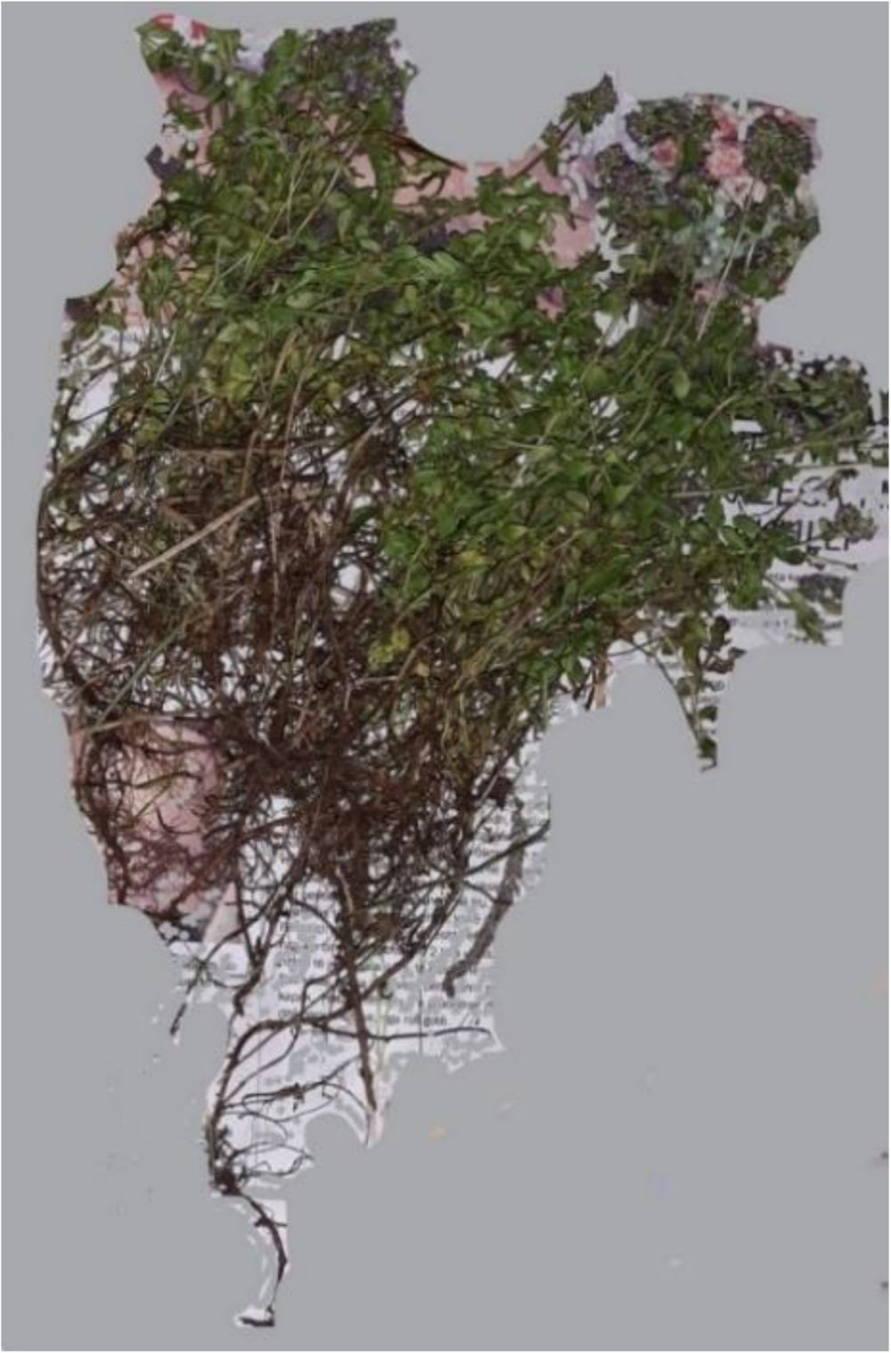
Thymus serpyllum.

*T. serpyllum* plants were collected in a field in Kosovo and preserved in an herbarium at the Biology Department of the University of Prishtina (Figure 1). The aerial portion of the plant was dried and ground to a powder with a blender. The dried powder was then vacuum packed and stored at –20°C until use.

**Figure 2:**
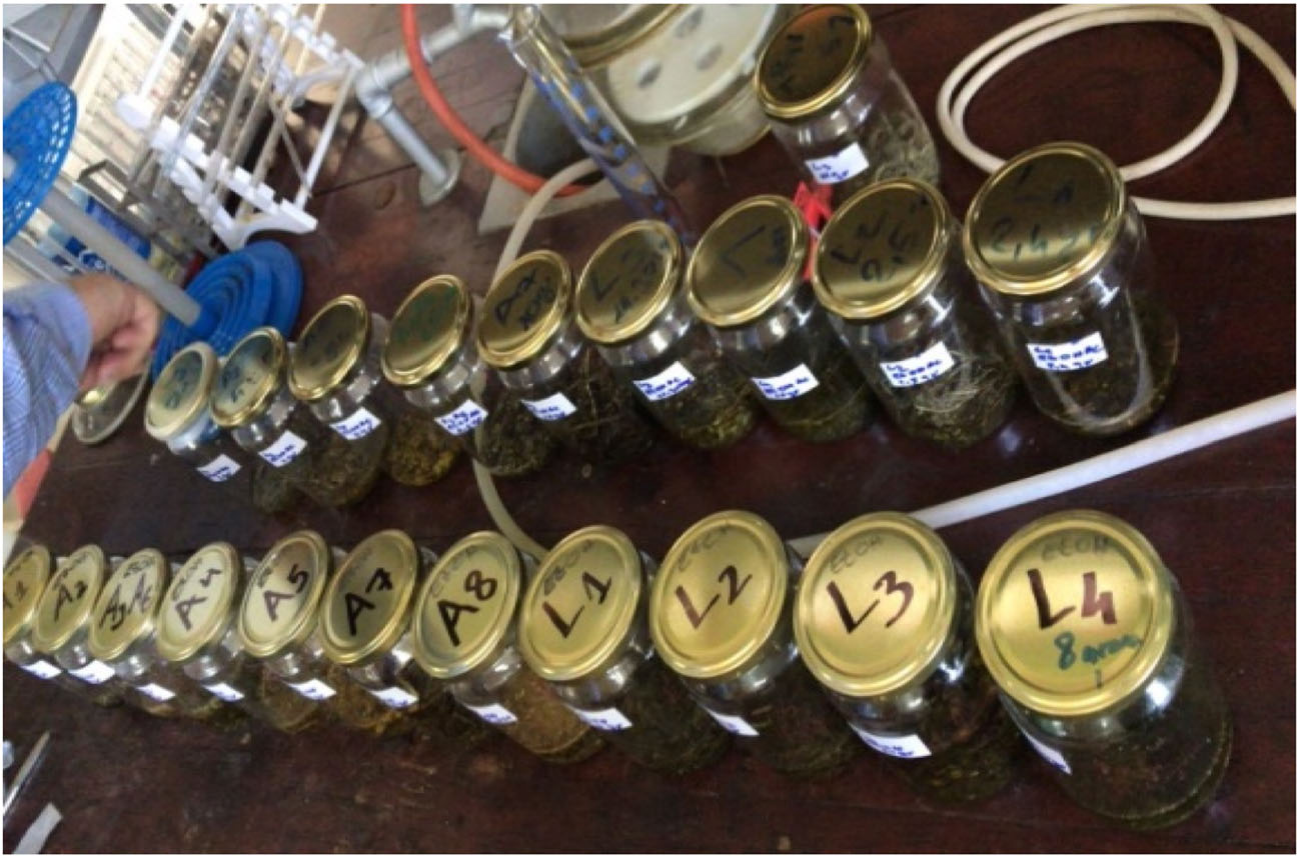
Herbal extraction.

We extracted 10 g of dried powder of *T. serpyllum* using 100 mL of 80% EthOAc with a Soxhlet apparatus, and then concentrated the extract with a rotary evaporator at 40°C (Figure 2). This extract was stored in a deep freezer at –20°C until the time of the experiment. For the fungicidal assays, the extracts were dissolved in DMF at a concentration of 100 mg/mL and stored at 4°C as a stock solution.

### 2.1 Fungicidal effect of *Thymus serpyllum*

The fungicidal effect of *T. serpyllum* extract, endodontic irrigant, and the combination of both was evaluated using *C. albicans* (ATTC 10,231, Liofilchem). These effects were analyzed using a disk diffusion assay (Jorgensen & Turnidge, 2007). This method in actual study consists of placing paper disks saturated with antifungal agents on a lawn of yeasts seeded on the surface of an agar medium, incubating the plate, and measuring the presence or absence of a zone of inhibition around the disks. The suspension of *C. albicans* was cultured for 48 h in 1 mL of sterile Sabouraud dextrose broth (Liofilchem) at 37°C, and then adjusted to a turbidity of 0.5 on the McFarland scale (1.5 × 108 cells/mL). After pouring 10 mL of Mueller–Hilton agar into Petri dishes, they were inoculated with yeast strains by adding of 0.1 mL of cell culture media, according to Kirby–Bauer technique (Hudzicki, 2016).

Sterile filter paper disks loaded with endodontic irrigants, plant extracts, and a combination of both (10 mg/mL) were placed on top of the agar plates, which were then incubated at 35°C for 24 h. DMF served as a positive control, while microorganisms alone served as a negative control group. According to the disk diffusion method, antimicrobial agent diffuses into the agar in Petri dishes and inhibits germination and growth of the tested microorganism. After this, the diameters of inhibition growth zones will be measured (Balouiri et al., 2016).

**Figure 3:**
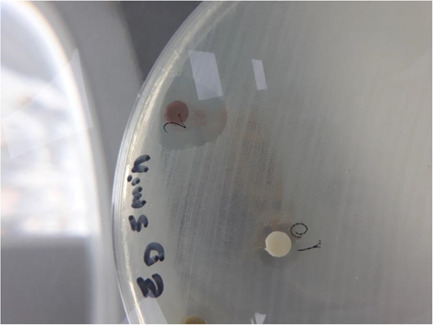
Observation of Inhibition zone (mm).

Inhibition zones, considered indicative of fungicidal activity, were measured using Vernier calipers and recorded (Figure 3).

### 2.2 Genotoxicity assay of *Thymus serpyllum*

An in vitro genotoxicity assay was performed in cultured lymphocytes. Blood (12 mL) was taken from 30 human volunteers. EthOAc extracts of *T. serpyllum* (0.5 mL) were evaluated in four groups. Glyphosate herbicide (36%) was used as a positive control, while no agent was used for the negative control. Blood (1 mL) from human volunteers was taken from heparinized venous blood vessels and transferred to 15‐mL tubes containing peripheral blood medium (Sigma) for lymphocyte cultivation (Fenech & Morley, 1986; Fenech, 1993). The tubes were then incubated for 72 h at 37°C.

After 44 h of incubation, 3 µg/mL of cytochalasin B was added according to the method of Fenech and Morley (Fenech & Morley, 1986; Fenech, 1993), which ensures cytokine production and produces binuclear cells within the parent cell. Cell cultures were then centrifuged for 10 min at 1000 rpm. The supernatant was discarded, and the pellet containing the lymphocytes was resuspended in 5 mL of hypotonic KCl solution (74 mM) that was prewarmed to 37°C before the cells were subjected to hypotonic shock for 10 min at room temperature. The cultures were then centrifuged for another 10 min at 1000 rpm. After removing the supernatant, the cells were fixed for 20 min with a fixative solution (3: 1 ratio of absolute ethanol: glacial acetic acid) that was precooled at 4°C. After centrifugation and fixation, the cells were finally resuspended in 1 mL of fresh fixation solution. The extract preparations were placed onto pre‐chilled glass slides, which was sufficient for the counting of 500 LBN‐binuclear lymphocytes. These preparations were dried at room temperature for several hours and then stained with 10% Giemsa solution for 20 min before being rinsed with distilled water.

The results were analyzed and compared using a one‐way analysis of variance (ANOVA) test for normally distributed groups or the Kruskal–Wallis test for non‐parametric groups (SPSS Statistics 22.0). A level of *p* < .05 was considered statistically significant.

## 3 RESULTS

The growth of *C. albicans* colonies was recorded at 5 min, 60 min, and 24 h after the addition of an EthOAc extract of *T. serpyllum‐L2(4) to* all samples tested with endodontic irrigates. We did not observe the inhibition zone for 5 min after combining *Thymus serpyllum* extract with NaOCl. When the extract was used alone, there was also no inhibition zone. However, combining the extract with CHX resulted in an inhibition zone of 29.7 mm, which was similar to CHX alone (30.3 mm). There was a significant difference at 5 min in the samples exposed to the combination of CHX with the plant extract compared with that seen in the other groups tested (Table 1, *p* = .001).

**Table 1:** Effect of EthOAc extract of Thymus serpillum irrigant in *Candida albicans* growth hours.

	5 min				60 min				24 h			
*N* = 3	Mean	SD	Min	Max	Mean	SD	Min	Max	Mean	SD	Min	Max
EthOAc	0.0	0.0	0	0	0.0	0.0	0	0	0.0	0.0	0	0
NaOCl/EthOAc	0.0	0.0	0	0	0.0	0.0	0	0	0.0	0.0	0	0
CHX/EthOAc	29.7	2.1	28	32	28.3	1.5	27	30	29.0	0.0	29	29
NaOCl	69.7	0.6	69	70	65.0	0.0	65	65	68.3	0.6	68	69
CHX	30.3	0.6	30	31	19.3	0.6	19	20	21.3	3.2	19	25
DMF	0.0	0.0	0	0	0.0	0.0	0	0	0.0	0.0	0	0
Kruskal−Wallis	*p* = .001				*p* = .001				*p* = .001			

Abbreviations: CHX, chlorhexidine; DMF, dimethylformamide; EthOAc, ethyl acetate; KW, Kruskal−Wallis; NAOCl, sodium hypochlorite.

There was also no inhibition zone seen 60 min after combining L2(4) with NaOCl or with the extract alone. Similar to that seen at 5 min, we noted that combining the extract with CHX resulted in an inhibition zone of 28.3 mm. This effect was more pronounced than what was seen with CHX alone (19.3 mm). There was a significant difference at 60 min in the samples that were incubated with a combination of CHX with the plant extract compared with that seen in the other groups tested (Table 1, *p* = .001).

Similar to the other time points, there was no inhibition zone 24 h after combining L2(4) with NaOCl or the extract alone. When we combined the extract with CHX, the inhibition zone was 29 mm, which was higher than that seen with CHX alone (21.3 mm). There was a significant difference at 24 h in the samples that were exposed to a combination of CHX with the plant extract compared with that seen in the other groups tested (Table 1, *p* = .001).

**Figure 4:**
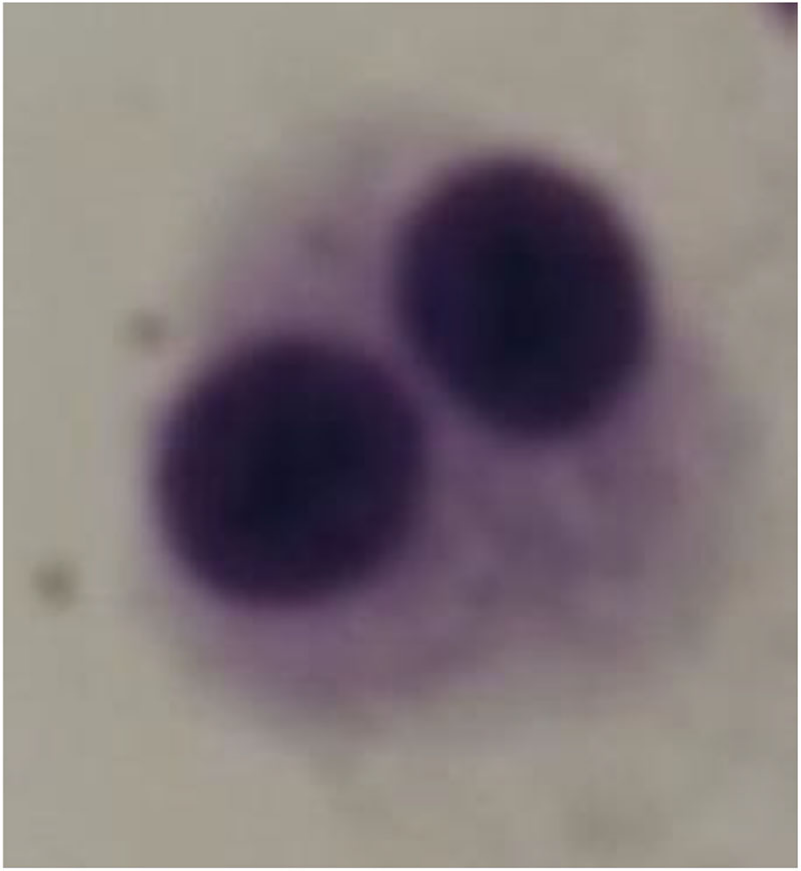
Binuclear lymphocytes with micronuclei in the group tested with glyphosate herbicide.

The genotoxic effects of EthOAc extracts of *T. serpyllum* (Figure 4) and glyphosate herbicide as a positive control were evaluated by observing the occurrence of micronuclei in 500 lymphocytes. There was a significance in the occurrence of micronuclei between glyphosate herbicide and EthOAc extracts (*p* = .000034) (Table 2).

**Table 2:** Genotoxic effects of glyphosate herbicide and thymus serpillum assessed by induction of micronuclei in human lymphocytes.

Agents (Mean%±SD) One‐way ANOVA		
Agent	Mean (% ±SD)	One way ANOVA
EthOAc extract	0	
Glyphosate herbicid	2.89 ± 1.21	*p* = .000034
No agent	0	

Abbreviation: EthOAc, ethyl acetate.

## 4 DISCUSSION

Herbal extracts are an attractive alternative therapeutic method against microbial‐infected root canal because of their minimal adverse effects, low cost, renewability, and are better tolerated by patients (Sainudeen et al., 2020). Active components in herbal extracts have antimicrobial, anti‐inflammatory, and antioxidant effects. For example, several studies have shown that thyme extracts have antimicrobial, anti‐inflammatory, and wound‐healing activities (Aazza et al., 2014; Ocaña & Reglero, 2012). Furthermore, numerous reports have validated the fungicidal activities in vitro of this essential oil on some human pathogens, including *C. albicans* (Gabriel et al., 2018; Parker et al., 2022). The antibacterial action of thymus oil is mainly due to its phenolic components, thymol and carvacrol (Jamali et al., 2012). Therefore, we felt that it would be useful to investigate this extract in our current study because there is limited evidence for its role as a root canal irrigant.

Fani and Kohanteb (2017) *reported that Thymus vulgaris was effective against C. albicans* isolated from patients with denture stomatitis and infected root canal in a zone of inhibition of 7.5‐42.00 mm in agar plates with *Thymus vulgaris* oil with a concentration between 156 and 256 µg/mL. Furthermore, Roxo et al. (2020) showed that this thymus oil was able to inhibit germ tube formation and biofilm integrity in *C. albicans*. Finally, Oliveira et al. (2017) demonstrated that *T. vulgaris* L. extract exhibited effective biological activities and could control monomicrobial biofilms of *C. albicans* when used at 200 mg/mL. Our study also revealed a strong inhibitory activity of *T. serpyllum extract against C. albicans after its combination with CHX*. This is likely a result of its hydrophobic properties that could disrupt the fungal structure (de Lira Mota et al., 2012).

In endodontics, the toxic effects of root canal irrigants and sealants have always presented challenges and have been a source of debate. Because micronuclei are strongly indicative of chromosomal aberrations, the micronuclei formation assay is a valid test for carcinogenicity (Luzhna et al., 2013). *T. vulgaris* has previously been shown to have no genotoxic and cytotoxic effect (de Lira Mota et al., 2012; Oliveira et al., 2017). Similarly, in our study, *T. serpyllum* treatment did not result in micronuclear cell formation in lymphocytes, suggesting that it is not genotoxic. The herbicide glyphosate was used in a control group and formed approximately 15 micronuclei in 500 observed lymphocytes (3.4%).

Together, it was shown that *T. serpyllum* has pronounced fungicidal activity with minimal genotoxic effects, suggesting that it may be an alternative root canal treatment irrigant. The addition of CHX further improved its antifungal effect against *C. albicans*. While further studies are needed to confirm its antimicrobial and genotoxic effects, we recommend that *T. serpyllum* extracts can be used in combination with CHX as an alternative disinfectant in root canals.

Based on numerous in vivo studies, root canal infections are invaded by polymicrobial species (Ricucci & Siqueira, 2010), who reported that biofilms were found mostly in root canals associated with apical periodontitis. This may be due to the fact that endodontic microbiota can be hidden in anatomical complexities, such as isthmuses, lateraly and accessory root canals, and so they can survive even after root canal disinfection (Vera et al., 2012).

The ability of root canal disinfection to dissolve and disrupt the microbial cell matrix is the key mechanism for antimicrobial agent (Wong et al., 2021).

So, the future studies should be focused on these agents abilities, to contribute for qualitative endodontic therapy and reduce the incidence of adverse clinical complications, such as apical periodontitis.

## 5 CONCLUSIONS

In conclusion, EthOAc extracts of *T. serpyllum* didn't show antifungal effect against *Candida Albicans* after combination with NaOCl; EthOAC extracts of *T. serpyllum* show antifungal effect against *C. Albicans* in combination with CHX, and can be used as an alternative disinfectant for root canal disinfection. Furthermore, this extract showed no genotoxic effects.

## AUTHOR CONTRIBUTIONS

All authors have made substantive contributions to this study and/or manuscript, and all have reviewed the final paper before its submission. Ariana Kameri contributed in study conception, manuscript preparation and data collection. Agime Dragidella contributed in manuscript preparation, Arben Haziri contributed in data analysis, Zeqir Hashani contributed in data collection and data interpretation, Kemajl Kurteshi contributed in data interpretation and Arsim Kurti contributed in data interpretation.

## CONFLICT OF INTEREST STATEMENT

The authors declare no conflict of interest.

## ETHICS STATEMENT

This research was approved by the Ethics Committee of the Medical Faculty in University of Prishtina and performed with the principles of medical ethics according to the Helsinki Declaration on Human Research. The patients signed a written informed consent form before the treatment.

## Data Availability

Data that support the findings of this study are available on request from the corresponding author.
